# Determinants of Smoking and Quitting in HIV-Infected Individuals

**DOI:** 10.1371/journal.pone.0153103

**Published:** 2016-04-21

**Authors:** Susan Regan, James B. Meigs, Steven K. Grinspoon, Virginia A. Triant

**Affiliations:** 1 Massachusetts General Hospital Division of General Internal Medicine and Harvard Medical School, Boston, Massachusetts, United States of America; 2 Massachusetts General Hospital Program in Nutritional Metabolism and Harvard Medical School, Boston, Massachusetts, United States of America; 3 Massachusetts General Hospital Divisions of General Internal Medicine and Infectious Diseases and Harvard Medical School, Boston, Massachusetts, United States of America; Institute of Infectious Disease and Molecular Medicine, SOUTH AFRICA

## Abstract

**Background:**

Cigarette smoking is widespread among HIV-infected patients, who confront increased risk of smoking-related co-morbidities. The effects of HIV infection and HIV-related variables on smoking and smoking cessation are incompletely understood. We investigated the correlates of smoking and quitting in an HIV-infected cohort using a validated natural language processor to determine smoking status.

**Method:**

We developed and validated an algorithm using natural language processing (NLP) to ascertain smoking status from electronic health record data. The algorithm was applied to records for a cohort of 3487 HIV-infected from a large health care system in Boston, USA, and 9446 uninfected control patients matched 3:1 on age, gender, race and clinical encounters. NLP was used to identify and classify smoking-related portions of free-text notes. These classifications were combined into patient-year smoking status and used to classify patients as ever versus never smokers and current smokers versus non-smokers. Generalized linear models were used to assess associations of HIV with 3 outcomes, ever smoking, current smoking, and current smoking in analyses limited to ever smokers (persistent smoking), while adjusting for demographics, cardiovascular risk factors, and psychiatric illness. Analyses were repeated within the HIV cohort, with the addition of CD4 cell count and HIV viral load to assess associations of these HIV-related factors with the smoking outcomes.

**Results:**

Using the natural language processing algorithm to assign annual smoking status yielded sensitivity of 92.4, specificity of 86.2, and AUC of 0.89 (95% confidence interval [CI] 0.88–0.91). Ever and current smoking were more common in HIV-infected patients than controls (54% vs. 44% and 42% vs. 30%, respectively, both P<0.001). In multivariate models HIV was independently associated with ever smoking (adjusted rate ratio [ARR] 1.18, 95% CI 1.13–1.24, P <0.001), current smoking (ARR 1.33, 95% CI 1.25–1.40, P<0.001), and persistent smoking (ARR 1.11, 95% CI 1.07–1.15, P<0.001). Within the HIV cohort, having a detectable HIV RNA was significantly associated with all three smoking outcomes.

**Conclusions:**

HIV was independently associated with both smoking and not quitting smoking, using a novel algorithm to ascertain smoking status from electronic health record data and accounting for multiple confounding clinical factors. Further research is needed to identify HIV-related barriers to smoking cessation and develop aggressive interventions specific to HIV-infected patients.

## Introduction

Smoking is highly prevalent among HIV-infected patients [1–6] and is strongly associated with increased prevalence of smoking-related chronic diseases.[5, 7, 8] Cardiovascular disease (CVD) risk, which is known to be heightened in HIV disease, [9–13] has been shown to decrease with increased time since quitting smoking in an HIV cohort.[14] Smoking-related characteristics, including degree of nicotine dependence,[15, 16] readiness to quit,[3, 15] and frequency of quit attempts,[15] have been explored for HIV-infected patients. HIV-infected patients have been cited as a high-priority group for intervention by a major tobacco guideline.[17] Understanding the impact of HIV and HIV-related parameters on smoking will help to develop smoking cessation strategies tailored to this group.

The challenge of obtaining reliable smoking data from electronic health record (EHR) data sources represents a barrier to studying smoking among HIV populations in clinical care.[18, 19] Natural language processing (NLP) tools have been developed to identify and classify smoking-related portions of text in medical records [20–22] and represent a novel approach to this problem. However, individual NLP classifications must be integrated to create a clinically meaningful smoking status for a patient at specific point in time that is appropriate for clinical research use.

We investigated smoking outcomes in a health care system-based longitudinal observational cohort of HIV-infected patients and matched controls. To determine smoking status in this large cohort, we developed and validated an algorithm to assign smoking status using NLP data. While current smoking prevalence has been demonstrated to be elevated among HIV-infected patients, it is unclear the extent to which this is due to greater smoking initiation or reduced smoking cessation among this group. We assessed whether HIV infection is independently associated with ever smoking and current smoking. In order to assess the effect of HIV status on smoking cessation, we also examined the outcome of current smoking in analyses limited to ever smokers (persistent smoking or failure to quit). We controlled for cardiovascular risk factors because they are elevated among patients with HIV and diagnosis with cardiovascular disease has been associated with smoking cessation. In addition, we controlled for mood disorders and schizophrenia with have been associated with high smoking prevalence and difficulty quitting.[23, 24] We then examined specific correlates of the three smoking outcomes within the HIV-infected group to assess whether HIV-related clinical characteristics, which have been associated with cardiovascular outcomes,[25–27] may impact likelihood of smoking and ability to quit. We sought to provide a comprehensive investigation of the impact of HIV on smoking behaviors, specifically examining whether HIV-related clinical characteristics affect smoking outcomes independently of potentially confounding clinical factors.

## Methods

### Patient population

The cohort comprised HIV-infected patients (cases) matched to HIV-uninfected patients (controls) on the basis of age, gender, race, and number of medical encounters in a 3:1 ratio. Data were obtained from the Partners HealthCare System (PHS) Research Patient Data Registry (RPDR), a comprehensive database of administrative, billing and electronic health record (EHR) information including inpatient and outpatient encounters for over 4.5 million patients. Patients were eligible to be included as cases if they received care at Brigham and Women’s Hospital or Massachusetts General Hospital between 2005 and 2007. HIV infection was determined by inpatient or outpatient diagnosis of HIV (International Classification of Diseases, 9th Revision, Clinical Modification [ICD-9-CM] codes 042 and all subtypes, V08, and corresponding electronic health record codes). Exclusion criteria for both groups included diagnosis of coronary heart disease (CHD) prior to 2008, age <18 years, and death prior to January 1, 2008. The study period spanned the time of the earliest documented clinical encounter through October 31, 2008. This study was approved by the Partners Human Research Committee. Informed consent of study subjects was not obtained. The IRB approval included a waiver of the requirement to obtain informed consent because the risk to study subjects, including risk to privacy, was deemed to be minimal, obtaining informed consent of study subjects was not feasible and the rights and welfare of the subjects would not be adversely affected by the waiver.

### Smoking NLP algorithm validation

We used an NLP tool [28] to scan free text portions of the medical record, identify portions of text, or “tokens,” that contain smoking-related information, and classify each token as indicating a non-smoker, current smoker or former smoker. The performance of the classifier in categorizing individual tokens has been validated previously.[28] However, a single patient’s medical record may contain multiple tokens with discrepant classifications. We applied an aggregation rule for combining token classifications to assign a smoking status to a patient for a given calendar year (S1 Text). To validate the full algorithm, a sample of 250 HIV cases and 250 controls were randomly selected from among those with NLP data available. For each calendar year from the patient’s first encounter to the last, the reviewer classified the patient’s smoking status for the period as smoker, nonsmoker or unknown. We calculated sensitivity, specificity and AUC comparing the NLP-based algorithm to the gold standard of clinician medical record review for ever versus never and current versus not current smoking. We assessed the performance of the algorithm by patient characteristics that might be expected to affect physician documentation (HIV status, gender, age, cardiovascular risk factors) as well as time (calendar year) and the number of tokens found. We compared AUC using a nonparametric test. [29]

### Covariate ascertainment

Data extracted from the RPDR included demographic data (age, gender and self-reported race), ICD-9 diagnostic codes, laboratory test results, medication prescriptions, and free text notes. Patients were classified as having hypertension, diabetes, dyslipidemia, coronary heart disease, depression, anxiety, bipolar disorder and schizophrenia if a relevant ICD-9 code was found (see Table 1 for specific codes). Patients were considered to have used pharmacotherapy for smoking cessation if a prescription for varenicline or an outpatient prescription for nicotine replacement therapy (NRT) was found. Inpatient NRT use was not included because is commonly used for temporary abstinence during hospitalizations. Because bupropion is indicated for both depression and smoking cessation, this medication was not considered a cessation aid. For cases, we obtained the most recent CD4 cell count and HIV RNA laboratory results. HIV RNA results are presented as percent detectable (≥400 copies/ml) versus not detectable, and among those with detectable results, the mean log-transformed HIV RNA.

**Table 1:** Patient Characteristics^*^.

	All (N = 12933)		NLP Available (N = 9783)		Validation Sample (N = 500)	
	HIV+	HIV-	HIV+	HIV-	HIV+	HIV-
N	3487	9446	2868	6915	250	250
Age, mean (SD)	44.5 (10.6)	43.2 (10.7)	44.7 (10.6)	43.3 (10.5)	44.4 (10.3)	43.4 (9.2)
Female gender, N (%)	1121 (32)	3511 (37)	956 (33)	2817 (41)	96 (38)	91 (36)
Race						
Caucasian, N (%)	1802 (52)	4565 (48)	1485 (52)	3293 (48)	122 (49)	107 (43)
African-American, N (%)	722 (21)	1955 (21)	598 (21)	1473 (21)	58 (23)	56 (22)
Hispanic, N (%)	622 (18)	1695 (18)	534 (19)	1364 (20)	51 (20)	62 (25)
Other/Unknown, N (%)	341 (10)	1231 (13)	251 (9)	785 (11)	19 (8)	25 (10)
Cardiovascular risk factor, N (%)	1880 (54)	4268 (45)	1669 (58)	3793 (55)	123 (49)	147 (59)
Hypertension, N (%)	975 (28)	2895 (31)	888 (31)	2595 (38)	59 (24)	93 (37)
Diabetes, N (%)	612 (18)	1325 (14)	538 (19)	1203 (17)	47 (19)	45 (18)
Dyslipidemia, N (%)	1394 (40)	2899 (31)	1264 (44)	2637 (38)	96 (38)	104 (42)
Mood disorder, N (%)	1481 (43)	3034 (32)	1361 (47)	2701 (39)	119 (48)	104 (42)
Depression, N (%)	1224 (35)	2247 (24)	1128 (39)	2025 (29)	101 (40)	77 (31)
Anxiety, N (%)	857 (25)	2094 (22)	801 (28)	1910 (28)	68 (27)	69 (28)
Bipolar disorder, N (%)	261 (7)	504 (5)	242 (8)	435 (6)	21 (8)	17 (7)
Schizophrenia, N (%)	102 (3)	245 (3)	94 (3)	199 (3)	7 (3)	13 (5)
Pharmacologic smoking cessation, N (%)	266 (8)	350 (4)	255 (9)	341 (5)	19 (8)	9 (4)
Nicotine replacement therapy use, N (%)	131 (4)	215 (2)	127 (4)	211 (3)	7 (3)	8 (3)
Varenicline use, N (%)	169 (5)	195 (2)	161 (6)	189 (3)	13 (5)	3 (1)
ART use, N (%)	1705 (49)	—	1523 (53)	—	139 (56)	—
CD4 cell count, mean (SD)	501 (326)	—	497 (327)	—	442 (316)	—
CD4 cell count <200/mm^3^, N (%)	330 (16)	—	292 (17)	—	34 (22)	—
HIV RNA (log-transformed), mean (SD)	8.6 (2.7)	—	8.5 (2.7)	—	8.5 (2.6)	—
HIV RNA <400 copies/ml, N (%)	2975 (85)	—	2432 (85)	—	210 (84)	—
Encounters/year, median (IQR)	6.3 (3.0–11.5)	6.5 (3.1–13.3)	6.8 (3.5–11.6)	6.6 (3.5–12.0)	7.1 (3.3–12.6)	6.6 (4.0–12.5)
Inpatient	0.1 (0–0.2)	0.1 (0–0.3)	0.1 (0–0.3)	0.1 (0–0.4)	0.1 (0–0.3)	0.1 (0–0.3)
Outpatient	6.0 (2.7–11.0)	5.9 (2.8–11.7)	6.4 (3.2–11.1)	6.1 (3.1–11.2)	6.4 (3.0–12.2)	6.3 (3.6–11.5)
Years in health care system, median (IQR)	8.4 (3.6–12.1)	7.7 (2.8–11.9)	8.9 (4.5–12.4)	9.2 (4.6–12.4)	8.4 (3.6–12.1)	9.0 (3.7–12.1)

* ICD codes: hypertension = 401.xx; diabetes = 250.xx; dyslipidemia = 272.xx; depression = 311.xx, 296.2, 296.3; anxiety = 300.xx; bipolar disorder = 296.0, 296.1, 296.4–296.8; schizophrenia = 295.xx. NLP = natural language processing; SD = standard deviation; ART = antiretroviral therapy; IQR = inter-quartile range

### Statistical analysis

We applied the NLP-based algorithm to the entire cohort of patients with NLP data available to obtain annual level (current vs. not) and patient level (ever vs. never) smoking status. We considered each patient’s most recent smoking status to be their current smoking status. We present prevalence of ever and current smoking as well as quitting. We assessed differences by HIV status using chi-squared tests.

We examined associations with smoking status in a series of generalized linear models with a log link function and robust standard errors, considering 2 outcomes: ever smoking (classified as a smoker during any year of observation) and current smoking (classified as a smoker at most recent observation). We created a cardiovascular risk factor index, an ordinal variable (range: 0–3) indicating the number of cardiovascular risk factors (including hypertension, hypercholesterolemia, and diabetes). We represented mood disorders as a dichotomous variable that was positive if a diagnosis of depression, anxiety, or bipolar disorder was present.

To explore the influence of HIV-infection on smoking cessation, we repeated the model with current smoking as the outcome, but limited the analysis to ever smokers. The outcome of this analysis can be interpreted as persistent smoking, or failure to quit.

All models included the cardiovascular risk and mood disorder variables plus schizophrenia while controlling for age (as a continuous variable), gender, and race (white vs. other). The models predicting persistent smoking also included a term for ever use of smoking cessation medication (varenicline or outpatient NRT). We constructed models for each outcome including HIV status as a correlate, and then repeated them for HIV cases only adding dichotomous variables for ever use of antiretroviral therapy (ART), CD4 cell count (<200 vs. ≥200) and HIV RNA (< 400 copies/ml vs. ≥400 copies/ml) at the most recent observation. For CD4 and HIV RNA, additional categories were created for patients with missing laboratory data. Sensitivity analyses were conducted substituting nadir CD4 for recent CD4 cell count, continuous HIV RNA (log transformed) for dichotomous HIV RNA, and duration of ART use for ever ART use. Additional analyses in the overall and HIV-only persistent smoking model were conducted limiting to patients with at least 12 months between the first and last smoking status. We present adjusted rate ratios (RR) and 95% confidence intervals (CI). All tests were 2-sided with P values <0.05 considered significant. All analyses were conducted in Stata *(StataCorp*, *2008*. *Stata Statistical Software*: *Release 10*. *College Station*, *TX*: *Stata Corporation*).

## Results

### Cohort characteristics

The overall cohort included 3487 HIV and 9446 control patients. NLP identified at least 1 smoking-related token for 2868 cases (82%) and 6915 controls (73%). Among those with >1 token available, the median time between the first and last observation was 55 months. Table 1 presents the demographic and clinical characteristics of the entire cohort, patients with NLP data available, and the validation sample randomly drawn from those with NLP data available.

### Smoking algorithm validation

Smoking status was ascertained by both the NLP-based algorithm and the medical record reviewer for 500 patients during a total 1591 patient years. For current smoking, the NLP-based algorithm had a sensitivity of 92%, specificity of 86% and AUC of 0.89 (95% CI 0.88–0.91). For ever smoking, the NLP-based algorithm had a sensitivity of 94%, specificity of 73% and AUC of 0.84 (95% CI 0.81–0.87). The performance of the NLP-based algorithm as compared to medical record review in specific subgroups of patients is presented in Table 2.

**Table 2:** Performance of NLP-based^††^ Algorithm by Patient Characteristic.

		Current smoking (by year)*				Ever smoking*			
		Sensitivity	Specificity	AUC‡	P value	Sensitivity	Specificity	AUC‡	P value
Entire validation sample		92.4	86.2	0.89 (0.88–0.91)		94.3	73.4	0.84 (0.81–0.87)	
									
HIV status	HIV -	91.1	86.5	0.89 (0.86–0.91)	0.670	94.2	74.8	0.85 (0.80–0.89)	0.606
	HIV +	93.2	85.9	0.90 (0.87–0.92)		94.3	71.4	0.83 (0.78–0.88)	
									
Gender	Male	91.0	85.0	0.88 (0.86–0.90)	0.075	94.9	74.5	0.85 (0.81–0.89)	0.490
	Female	94.5	87.6	0.91 (0.89–0.93)		93.1	71.7	0.82 (0.77–0.88)	
									
Age	<45	92.7	87.6	0.90 (0.88–0.92)	0.212	93.3	73.8	0.84 (0.80–0.87)	0.443
	≥45	91.8	84.2	0.88 (0.85–0.91)		94.1	77.7	0.86 (0.81–0.91)	
									
Number of CVD risk factors†	0	92.8	87.3	0.90 (0.87–0.93)	0.535	91.7	76.1	0.84 (0.79–0.89)	0.948
	≥1	92.2	85.7	0.89 (0.87–0.91)		96.3	71.0	0.84 (0.79–0.88)	
									
Calendar year	<2005	92.9	88.2	0.91 (0.88–0.93)	0.199	91.2	82.9	0.87 (0.83–0.91)	0.183
	≥2005	92.0	84.8	0.88 (0.86–0.91)		91.8	75.5	0.84 (0.80–0.87)	
									
Number of NLP†† tokens	1	85.7	89.8	0.88 (0.84–0.91)	0.979	82.5	86.5	0.85 (0.77–0.92)	0.434
	2–5	92.0	84.2	0.88 (0.86–0.91)		91.6	79.8	0.86 (0.80–0.91)	
	>5	99.2	77.5	0.88 (0.84–0.93)		100.0	62.5	0.81 (0.77–0.86)	

* Smoking status by year is ascertained as current smoker versus nonsmoker. Smoking status by patient is ascertained as ever versus never smoker.

† CVD = cardiovascular disease; CVD risk factors include hypertension, diabetes, and dyslipidemia.

‡AUC = area under ROC curve.

††NLP = natural language processing.

### Prevalence and correlates of smoking

Using the NLP-based algorithm, overall smoking prevalence was 47% for ever smoking and 33% for current smoking. Smoking was more prevalent in HIV-infected patients compared to controls (54% vs. 44% for ever smoking, 42% vs. 30% for current smoking, P<0.001 for both comparisons). Persistent smoking (among ever smokers) was documented in 71% of the overall group, 77% of the HIV-infected patients, and 68% of the control patients. NRT was used by 7% (N = 333) of ever smokers, with no difference between cases and controls (8% vs. 7%, p = 0.111).

In multivariate modeling adjusted for age, gender, race, cardiovascular risk index, mood disorder, and schizophrenia, HIV infection was significantly associated with ever smoking (RR 1.18, 95% CI 1.13–1.24, P<0.001), current smoking (RR 1.33, 95% CI 1.25–1.40, P<0.001), and persistent smoking (RR 1.11, 95% CI 1.07–1.15, P<0.001).(Table 3) Male gender and being diagnosed with schizophrenia were the only other factors to show this consistent pattern across all three outcomes. The number of cardiovascular risk factors diagnosed was not associated with ever smoking, but each additional diagnosis was associated with a 10% decrease in the prevalence of current smoking (RR 0.91, 95% CI 0.88–0.94, P<0.001) and a 10% increase in quitting (RR 0.91 for persistent smoking, 95% CI 0.89–0.93, P<0.001). The presence of a mood disorder was associated with ever and current smoking but not with quitting smoking.

**Table 3:** Correlates of Smoking in Overall Group^*^.

	Ever Smoking			Current Smoking			Persistent Smoking		
	All Patients			All Patients			Ever Smokers		
	(N = 9783)			(N = 9783)			(N = 4601)		
	RR*	95% CI	P value	RR*	95% CI	P value	RR*	95% CI	P value
HIV-infected	1.18	1.13–1.24	<0.001	1.33	1.25–1.40	<0.001	1.11	1.07–1.15	<0.001
Age (decades)	1.00	0.98–1.02	0.877	1.01	0.98–1.04	0.465	1.01	0.99–1.03	0.291
Female	0.89	0.85–0.93	<0.001	0.77	0.72–0.82	<0.001	0.86	0.82–0.89	<0.001
White	1.02	0.97–1.06	0.462	1.03	0.97–1.09	0.298	1.02	0.98–1.06	0.363
Cardiovascular risk factors (0–3 ordinal)	1.00	0.98–1.03	0.689	0.91	0.88–0.94	<0.001	0.91	0.89–0.93	<0.001
Mood disorder (any, dichotomous)	1.45	1.39–1.51	<0.001	1.44	1.36–1.52	<0.001	0.98	0.94–1.02	0.272
Schizophrenia	1.24	1.14–1.35	<0.001	1.38	1.23–1.54	<0.001	1.09	1.01–1.18	0.035
Smoking cessation medication	—	—	—	—	—	—	1.36	1.30–1.48	<0.001

* RR = relative risk

In analyses repeated within the HIV-infected group only, having a detectable recent HIV RNA was significantly associated with ever smoking, current smoking, and persistent smoking (Table 4). Having a CD4 cell count less than 200/mm^3^ was associated with being less likely to quit smoking, although this association did not achieve statistical significance. The performance of the other factors followed a similar pattern between the HIV-infected only and overall models.

**Table 4:** Correlates of Smoking in HIV Patients.

	Ever Smoking			Current Smoking			Persistent Smoking		
	All HIV-Infected Patients			All HIV-Infected Patients			Ever Smokers		
	(N = 2868)			(N = 2868)			(N = 1558)		
	RR*	95% CI	P value	RR*	95% CI	P value	RR*	95% CI	P value
Age (decades)	1.02	0.99–1.06	0.171	1.01	0.97–1.06	0.521	0.99	0.96–1.02	0.526
Female	0.94	0.87–1.01	0.083	0.84	0.77–0.93	0.001	0.88	0.83–0.94	<0.001
White	1.10	1.02–1.17	0.010	1.13	1.04–1.24	0.006	1.05	0.99–1.11	0.087
Cardiovascular risk factors (0–3 ordinal)	0.96	0.93–1.00	0.038	0.87	0.83–0.91	<0.001	0.90	0.87–0.93	<0.001
Mood disorder (any, dichotomous)	1.39	1.29–1.49	<0.001	1.39	1.28–1.52	<0.001	0.99	0.94–1.05	0.814
Schizophrenia	1.17	1.02–1.33	0.028	1.16	0.96–1.40	0.132	0.98	0.86–1.12	0.777
ART† use (ever vs. never)	1.02	0.94–1.11	0.638	1.04	0.94–1.15	0.462	1.01	0.95–1.07	0.746
CD4 cell count <200/mm^3^	1.04	0.93–1.15	0.502	1.09	0.96–1.24	0.190	1.07	0.99–1.15	0.068
HIV RNA >400 copies/ml	1.13	1.04–1.24	0.008	1.25	1.12–1.41	<0.001	1.12	1.05–1.20	0.001
Smoking cessation medication	—	—	—	—	—	—	1.33	1.26–1.41	<0.001

*RR = relative risk

†ART = antiretroviral therapy

In further sensitivity analyses among the HIV-infected patients, we investigated the effects of CD4 cell count nadir, HIV RNA expressed as a continuous variable, and ART duration on the three outcomes in order to assess different aspects of HIV disease severity. Results were similar, with the exception of ART duration which was significantly associated with history of ever smoking (RR 1.02, 95% CI 1.01–1.03, P<0.001).

## Discussion

In a large clinical care cohort of HIV-infected and matched control patients, we found HIV to be a significant correlate of current and ever smoking with an effect size comparable to that for associations of smoking with male gender or schizophrenia, while controlling for cardiovascular risk factors and mental health disorders. We also showed being HIV infected to be independently associated with decreased likelihood of quitting smoking. Despite extensive data supporting a high prevalence of smoking among HIV-infected individuals, whether HIV infection is independently associated with smoking after accounting for multiple potentially confounding clinical factors has not been clearly established. Our finding that HIV infection is independently associated with both smoking and decreased likelihood of quitting strongly establishes HIV-infected patients as an extremely high-risk group meriting targeted smoking cessation intervention.

HIV-infected patients demonstrate extremely high smoking prevalence across multiple geographic and clinical settings, with a recent study demonstrating higher attributable mortality to smoking than to HIV itself.[30] Smoking prevalence among HIV-infected patients has ranged from 43% to 64% in a series of earlier cohort studies, [1–4, 15, 31] and was higher relative to matched control patients in French and Danish cohorts. [30, 32] A recent study comparing smoking prevalence in HIV-infected patients versus the general US population found current smoking prevalence of 42% for HIV compared with 21% for the general US population.[6] Our findings, which showed smoking prevalence of 42% versus 30% for HIV-infected compared with matched control patients in longitudinal clinical care, are highly consistent with results from this national cross-sectional survey.

HIV-infected patients were also found to be significantly less likely to quit smoking, despite higher prevalence of pharmacologic smoking cessation aids. Prior studies have reported relatively high motivation to quit smoking among HIV-infected patients [15] and high rates of quit attempts.[15] Yet this apparent readiness does not appear to translate into successful smoking cessation, as demonstrated in a recent study.[6] Several smoking cessation trials utilizing intensive counseling and cellular telephone interventions have demonstrated efficacy, [33–35] but were limited by short follow-up or non-randomized design. A recent study demonstrated increased smoking cessation rates following implementation of a training program for HIV clinicians.[36] Our findings reinforce the need for studies of intensive yet feasible smoking cessation interventions tailored to HIV-infected patients that can be readily applied within current care models.

Within the group of HIV-infected patients, we conducted several analyses exploring factors associated with smoking and smoking cessation to identify HIV subgroups that might be targeted for more intensive intervention. Patients with a detectable HIV viral load were significantly more likely to smoke and less likely to quit compared to those who were virologically suppressed, even after accounting for the presence of mental health disorders. While CD4 cell count was not associated with ever or current smoking, having a low CD4 cell count tended to be associated with not having quit smoking (P = 0.068). Importantly, these HIV-related factors appear to be more important in predicting patients’ ability to quit smoking than mood disorders or schizophrenia, which were not significant risk factors. The group of patients with less well controlled HIV infection might represent those not yet meeting previous criteria for antiretroviral treatment (as guidelines recommending treatment for all HIV-infected patients are relatively recent [37]) or those who are not adhering to prescribed therapy. Socio-demographic and clinical factors which affect medication adherence and lead to detectable viral load measurements might also represent barriers to smoking cessation.

Our findings are consistent with established risk factors for smoking in the general population, in which smoking prevalence is typically higher in men [38] and in patients with psychiatric disorders, [39] identified as a high risk group. [40] The presence of HIV infection coupled with a psychiatric disorder is likely to confer a heightened risk of smoking, given individual increased risks of 30% conferred by HIV infection, 25% by a mood disorder, and 20% by schizophrenia. This subgroup of HIV-infected patients with mental illness represents a particularly high risk group for whom aggressive smoking cessation intervention is warranted.

To optimize smoking data for our cohort, we developed and validated a novel algorithm to identify smoking status from EHR data using NLP. Several NLP tools for smoking status have been developed [20–22] and used to assess physician adherence to evidence-based guidelines [41] or to assign smoking status on the patient level [19]. Our purpose was to develop a method to use NLP token classifications in a way that reflected the longitudinal nature of our cohort and that could capture changes in smoking status over time. The algorithm performed extremely well, yielding sensitivity and specificity in the 90 percent range for annual smoking prevalence. Moreover, the algorithm performance remained consistent when evaluated according to multiple characteristics reflecting variation in patient characteristics and clinical care delivery.

The study was limited by several factors intrinsic to observational data. It was a retrospective observational study and therefore potentially subject to confounding, despite the demographic matching of the control group. We were unable to control for socioeconomic status and other substance use, variables likely to influence smoking behavior that might differ between HIV-infected patients and controls. The validation study was conducted using detailed medical record data by a trained clinical research nurse, as patient self-reported smoking data were not available. Our algorithm was by necessity validated in the cohort in which it was derived, rather than an external validation cohort, because the NLP tool we used was developed for and is specific to the Partners HealthCare System. While the algorithm we developed is applicable to EHR data in the Partners HealthCare System. The process by which it was generated is applicable to other health care systems, in which it might serve as a model for the development of analogous algorithms.

The implications of the study extend to both HIV management care and HIV clinical research methodology. We demonstrated that HIV is independently and significantly associated with history of smoking, current smoking, and decreased likelihood of quitting smoking. Additionally, we show that having less well-controlled HIV disease represents a barrier to quitting smoking with a stronger association than having a mental health disorder. Moreover, the development of an automated algorithm to identify smoking status from EHR data represents an innovative approach which can be translated to other settings and serve as a paradigm in a research era of increasing reliance on clinical care data. By substantiating the link between HIV infection and smoking and identifying HIV subgroups with lower likelihood of quitting, the data from this study provide strong support for intensifying efforts at the provider and public health level for HIV-specific smoking cessation strategies.

## Supporting Information

S1 Text Token Aggregration Rule Selection. (DOCX)
