# Supplementary material for: Determinants of Smoking and Quitting in HIV-Infected Individuals
Source: PLoS One. 2016 Apr 21;11(4):e0153103. doi: 10.1371/journal.pone.0153103 (PMC4839777; doi:10.1371/journal.pone.0153103)
Supplement: S1 Text — (DOCX) [file pone.0153103.s001.docx]

**S1 Text. Token Aggregration Rule Selection**

The NLP classifier assigns a smoking status (current smoker, past smoker, or nonsmoker) to each token found in a patient’s medical record. We wished to combine the individual token classifications into a patient-level smoking status during a fixed interval, i.e. a year. Because each token was derived from a dated note in the medical record, we could group each patient’s tokens by calendar year. We then counted the number of tokens within each patient/year that were classified as current smoker, past smoker, and nonsmoker. We considered 6 alternative rules for assigning a dichotomous smoking status (current smoker vs not current smoker) for the year (Supplemental table).

To assess the rules, we divided all the tokens into patient/years and randomly selected 1000 patient/years for validation. A human reader was used as a gold standard. The human reader read all the tokens for a patient/year and assigned the patient a smoking status for the year. The human reader remained blind to the NLP classifications of the tokens. Patient/years in which the human reader could not determine smoking status were excluded. The human classifications were compared to those of each rule, and the sensitivity, specificity and AUC of each rule was calculated (Supplemental Table 1). We chose Rule 6, a patient is a smoker if there are more current smoker tokens than nonsmoker tokens. This rule had the greatest AUC and sensitivity and specificity values that were high and approximately equal to one another.

**Supplemental Table 1. Performance of token aggregation rules in assigning smoking status to a patient/year.**

| Measure | Patient is a Current Smoker if ... | | | | | |
| --- | --- | --- | --- | --- | --- | --- |
|  | *Rule 1* | *Rule 2* | *Rule 3* | *Rule 4* | *Rule 5* | *Rule 6* |
|  | Smoker>0  OR Past>0 | (Smoker>0 OR Past>0) AND Nonsmoker=0 | Smoker>0 | Smoker>0 AND Nonsmoker=0 | Smoker>  (Nonsmoker + PastSmoker) | Smoker>Nonsmoker |
| Sensitivity (%) | 98 | 84 | 93 | 79 | 83 | 89 |
| Specificity (%) | 67 | 84 | 81 | 92 | 92 | 90 |
| AUC (95% CI) | .83  (.81-.84) | .84  (.82-86) | .87  (.85-.89) | .87  (.84-.88) | .88  (.86-.89) | .90  (.88-.91) |

Smoker = number of current smoker tokens; Past = number of past smoker tokens; Nonsmoker= number of nonsmoker tokens

AUC = area under ROC curve; CI = confidence interval.
